# Supplementary material for: Lactic acid bacterium F. sanfranciscensis impairs fitness of yeast M. humilis in a synthetic wheat sourdough medium
Source: Appl Environ Microbiol. 2026 Sep 17;92(10):e01030-26. doi: 10.1128/aem.01030-26 (PMC13688844; doi:10.1128/aem.01030-26)

Gating strategy

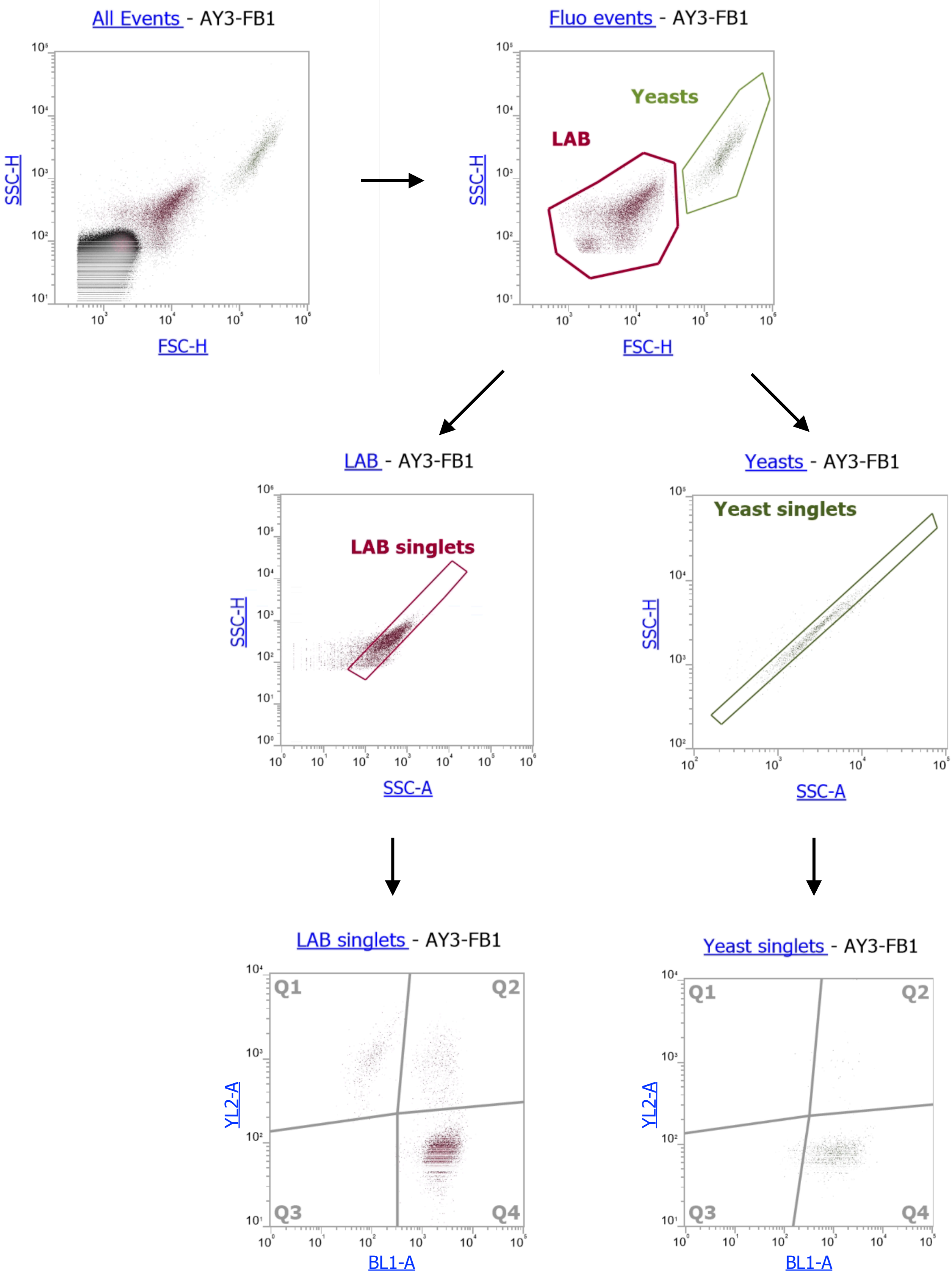

Supplement: Supplementary file 1 [file aem.01030-26-s0001.pdf]
